# COVID-19, severe mental illness, and family violence

**DOI:** 10.1017/S0033291721000490

**Published:** 2021-03-12

**Authors:** Emilie K. Wildman, Deirdre MacManus, Elizabeth Kuipers, Juliana Onwumere

**Affiliations:** 1 Department of Psychology, King's College London, Institute of Psychiatry, Psychology & Neuroscience, London, UK; 2 Department of Forensic and Neurodevelopmental Sciences, King's College London, Institute of Psychiatry, Psychology & Neuroscience, London, UK; 3 Bethlem Royal Hospital, South London and Maudsley NHS Foundation Trust, Beckenham, UK

‘*For as long as we allow family violence to remain in the shadows, it will do just that – remain*’ (Campbell, 2020)

## COVID-19 and the implementation of public health measures

On 11th March 2020, the World Health Organisation (WHO) announced that the COVID-19 virus was a pandemic. Subsequently, a wave of dedicated measures was implemented by countries around the world in an effort to slow its spread. Closure of schools and universities, remote working, travel restrictions, home confinement and ‘social’ (i.e. physical) distancing were among the key preventative measures that were adopted within the United Kingdom (UK) and many other countries.

Mounting concern about how the pandemic, and the corresponding public health measures, might affect the mental health of the general population, prompted rapid research into the potential effects of COVID-19 on mental health. In the UK general population, evidence covering the period from October 2019 to April 2020 suggests that, when compared to data from the final quarter of 2019, adults have reported significantly greater levels of anxiety and lower levels of positive mental wellbeing in the first quarter of 2020 (ONS, 2020). Evidence further suggests that the largest deterioration can be observed in clinical populations; those with the poorest mental health prior to the pandemic (Xu & Banks, 2020). However, compared to the general population, the needs of clinical populations such as those with severe mental illness (SMI) have received less attention (The Lancet Psychiatry, 2020). Since COVID-19 and the measures introduced to slow its spread have been shown to be associated with higher stress and an observable deterioration in the mental health of the general population (Xu & Banks, 2020), it is possible that these effects are likely to be even greater or more challenging in populations with SMI.

## COVID-19: increasing the risk of family violence

The COVID-19 pandemic and consequent public health measures have shone a sharp light on inter-personal relationships, including issues of family violence (Campbell, 2020; Refuge, 2020; Usher, Bhullar, Durkin, Gyamfi, & Jackson, 2020). Parallel to concerns about the impact of COVID-19 on mental health are increased concerns about rising rates of domestic violence, following the implementation of measures such as enforced home confinement (Campbell, 2020). Evidence to date suggests that stay at home orders may increase the vulnerability of victims of family violence (Campbell, 2020; Usher et al., 2020), which is likely to also extend to settings and relationships where family violence is perpetrated by an individual with SMI.

Pre-COVID-19, available evidence estimated the lifetime prevalence rate of family violence perpetration by individuals with SMI to be 50–60% (Kageyama et al., 2015). However, researchers suggest that rates are likely to be higher, since family violence perpetrated by individuals with SMI often goes unreported (Onwumere, Parkyn, Learmonth, & Kuipers, 2019). Family members' fears of stigmatising and adversely affecting the care of relatives with SMI, are commonly cited factors that tend to deter them from disclosing to others the ‘secret’ of being the target of violence from their relative (Kageyama et al., 2015; Onwumere et al., 2019). Elevated stress levels, co-residency and increased family contact are all known risk factors for those with SMI to perpetrate violence towards family members who are, themselves, more likely to be victims of their violence when compared to all other groups (e.g. general public) (Kageyama et al., 2015). These risk factors are likely to be exacerbated by COVID-19. As previously highlighted, evidence shows that the impacts of COVID-19 include heightened anxiety and stress levels, and that this is particularly marked in populations with SMI (Xu & Banks, 2020). Moreover, public health measures such as stay at home orders, which have forced families together, inevitably increase family contact hours. Under these conditions, the risk of family violence perpetration by individuals with SMI is likely to increase.

## Responding to family violence by individuals with SMI during COVID-19: an urgent call for increased research efforts and service adaptations

Stay at home orders present unique challenges for those family members who are unsafe at home, and the services designed to support such families. Home confinement is likely to limit the ability of healthcare providers and other agencies to accurately identify and respond to family violence by those with SMI. It will also limit the accessibility of support options for those living with and impacted by family violence (Usher et al., 2020). We know the effects of family violence are rarely limited to the identified victim(s) and perpetrator, but will also be felt by the wider family network. Support interventions designed to address family violence require a broader approach that offers flexibility to also address the needs presented by the wider family network (Tiyyagura et al., 2020). As the pandemic continues and countries that are significantly affected introduce different variations of local, regional and national lockdowns, healthcare providers are likely to benefit from additional resources, including specific training packages, that help to facilitate the accurate, timely and sensitive identification, and provision of support for families affected by violence committed by a relative with SMI. For example, healthcare workers should receive regular training that aims to: (a) increase awareness about different types of violence that can occur in family relationships, including psychological and emotional types, which may not carry visible signs of harm; (b) promote a readiness and confidence in enquiring about violence perpetration, and using discreet methods of enquiry when appropriate (e.g. use of closed questions which can be responded to with non-verbal signals such as nodding or coughing) and; (c) improve confidence in responding to disclosures of violence and awareness of relevant referral, service, and support pathways; an approach likely to increase the effectiveness of signposting and potential inter-agency working. This will also include improving mental health services' responses to individuals with SMI that perpetrate family violence (Bhavsar, Kirkpatrick, Calcia, & Howard, 2020), and will likely involve adapting and optimising existing channels of support. Digitalised and telephone-based support might be helpful for physically and socially isolated families. They might also offer more immediately accessible and discreet avenues of support for family member victims, who remain at home with their relative with SMI during COVID-19. Families with limited access to digital channels might be prioritised for more outreach or clinic-based interventions. The wider dissemination of alternative support resources, through primary care settings such as GP surgeries and pharmacies, might also be of benefit.

It is unfortunate that family experiences during COVID-19 have largely been neglected in the research literature. Research utilising qualitative and quantitative methods that focus on establishing the support needs of families during COVID-19 and how these are specifically impacted within the context of severe mental health problems and violence, are recommended.

## Conclusion

Prioritising the needs of families at the intersection of SMI and violence has long been neglected within research, clinical and policy domains. The lack of commentary on COVID-19 and family violence, specifically pertaining to contexts in which the perpetrator has an SMI, continues to represent a limitation within domestic violence research. If we are to remain committed to improving outcomes for families affected by severe mental health problems and violence, increased efforts should be directed towards supporting further research and service developments. Although this editorial has focused its attention on family violence perpetrated by individuals with SMI, the issue of violence by family members towards relatives with SMI, bidirectional violence or family violence, *per se*, must not be overlooked.
